# Supplementary material for: CCN1/Integrin α5β1 Instigates Free Fatty Acid-Induced Hepatocyte Lipid Accumulation and Pyroptosis through NLRP3 Inflammasome Activation
Source: Nutrients. 2022 Sep 19;14(18):3871. doi: 10.3390/nu14183871 (PMC9505842; doi:10.3390/nu14183871)
Supplement: Supplementary file 1 [file nutrients-14-03871-s001.zip › nutrients-1869266-supplementary.pdf]

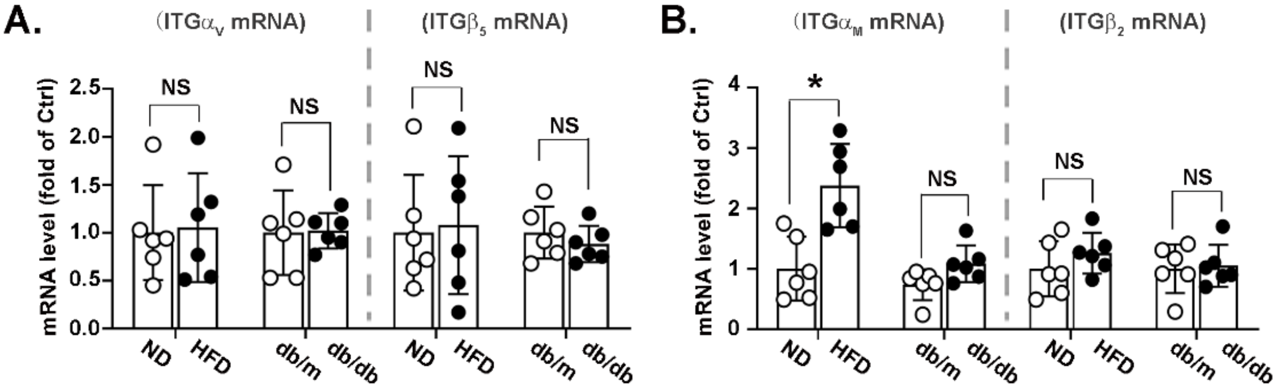

Figure S1. Expression of integrin $\alpha_V\beta_5$ and $\alpha_M\beta_2$ in the livers of obese mice. Mice fed a ND or HFD for 16 weeks (n=6 in each group) and db/db and db/m mice (8 weeks old, n=6 in each group) were sacrificed. The livers were immediately dissected and subjected to quick-freezing in liquid nitrogen. The mRNA levels of integrin $\alpha_V\beta_5$ **(A)** and $\alpha_M\beta_2$ **(B)** were measured by RT-qPCR. $^*p<0.05$.

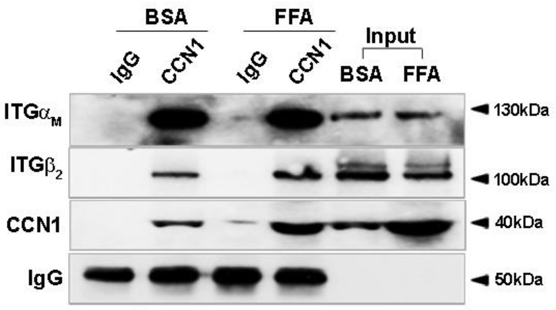

Figure S2. Effects of FFA on binding of CCN1 to integrin $\alpha_M\beta_2$. Immunoblotting (integrin α_M and β_2) of whole cell lysates (input) and immunoprecipitates (CCN1) from LO2 hepatocytes with or without FFA (1 mM) treatment for 24 h. Immunoblots shown are representative of three independent experiments.
